# A new virus found in garlic virus complex is a member of possible novel genus of the family *Betaflexiviridae* (order *Tymovirales*)

**DOI:** 10.7717/peerj.6285

**Published:** 2019-01-16

**Authors:** Leonardo A. Da Silva, Athos S. Oliveira, Fernando L. Melo, Daniel M.P. Ardisson-Araújo, Francisco V. Resende, Renato O. Resende, Bergmann M. Ribeiro

**Affiliations:** 1 Department of Cell Biology/Institute of Biological Sciences, University of Brasilia, Brasilia, Distrito Federal, Brazil; 2 Department of Biochemistry and Molecular Biology, Federal University of Santa Maria, Santa Maria, Rio Grande do Sul, Brazil; 3 Embrapa Hortaliças, Empresa Brasileira de Pesquisa Agropecuária, Brasilia, Distrito Federal, Brazil

**Keywords:** GYMaV, Garlic, Virus complex, Betaflexivirus, Genome sequencing, *Betaflexiviridae*

## Abstract

Plant vegetative propagation strategies for agricultural crops cause the accumulation of viruses, resulting in the formation of virus complexes or communities. The cultivation of garlic is based on vegetative propagation and more than 13 virus species from the genera *Potyvirus*, *Allexivirus* and *Carlavirus* have been reported. Aiming for an unbiased identification of viruses from a garlic germplasm collection in Brazil, total RNA from eight garlic cultivars was sequenced by high-throughput sequencing (HTS) technology. Although most viruses found in this study were previously reported, one of them did not belong to any known genera. This putative new virus was found in seven out of eight garlic cultivars and phylogenetic data positioned it as representative of an independent evolutionary lineage within family *Betaflexiviridae*. This virus has been tentatively named garlic yellow mosaic-associated virus (GYMaV), sharing highest nucleotide identities with African oil palm ringspot virus (genus *Robigovirus*) and potato virus T (genus *Tepovirus*) for the replicase gene, and with viruses classified within genus *Foveavirus* for the coat protein gene. Due to its high frequency in garlic cultivars, GYMaV should be considered in upcoming surveys of pathogens in this crop and in the development of virus-free garlic plants.

## Introduction

Garlic (*Allium sativum* L.) is one of the most consumed vegetables in the world with triennial world production (2011–13) of over 23 million tons (Camargo-Filho & Camargo, 2015). Since garlic cultivation is based on vegetative propagation, viruses can accumulate after successive planting cycles and spread to different regions by contaminated bulbs (Conci, Canavelli & Lunello, 2003). To date, many viral diseases have been reported, some of which have devastating effects on garlic development (Conci, Canavelli & Lunello, 2003; Lunello, Di Rienzo & Conci, 2007). Garlic plants infected with the so-called “virus complex” (VC), which includes mainly viruses from the genera *Potyvirus*, *Carlavirus*, and *Allexivirus*, have significantly reduced bulb weight and perimeter (Lunello, Rienzo & Conci, 2007).

In these VCs, garlic viruses A-to-D (GVA, GVB, GVC, and GVD), garlic virus X (GVX), and garlic mite-borne mosaic virus (GMbMV) (Bereda, Paduch-Cichal & Dabrowska, 2017; Mituti et al., 2015; Wylie et al., 2014; Ardisson-Araújo et al., 2013) are often reported. These allexiviruses are transmitted by eriophyid mites (Kang et al., 2007). However, as for most of the viruses found in such complexes, their worldwide spread is generally due to the transportation of bulbs or other plant parts for vegetative propagation with no phytosanitary inspections. Other examples of viruses often identified in VCs are potyviruses transmitted by aphids such as garlic mosaic virus (GMV), leek yellow stripe virus (LYSV) and onion yellow dwarf virus (OYDV) (Fajardo et al., 2001; Mituti et al., 2015; Wylie et al., 2014) and the carlaviruses garlic latent virus (GLV), garlic common latent virus (GCLV) and shallot latent virus (SLV) (Tsuneyoshi et al., 1998). Besides these viruses with positive single-stranded RNA genomes, iris yellow spot virus (IYSV) (family *Tospoviridade*) has drawn attention for infecting both garlic and onion (Bag et al., 2015). IYSV isolates have a segmented negative single-stranded RNA genome and are transmitted by thrips (Turina, Kormelink & Resende, 2016).

In this study, we identified viruses present in different garlic cultivars from the germplasm collection of EMBRAPA Hortaliças, Brazil. The majority of the viruses found in these samples were previously reported, except for a new virus putatively classified as a member of new genus in the family *Betaflexiviridae* (order *Tymovirales*).

## Materials & Methods

### Garlic samples

The eight garlic cultivars analyzed in this study are part of the germplasm collection of the Brazilian Agricultural Research Corporation on Vegetables (EMBRAPA Hortaliças), Brazil. These cultivars are known as *Branco Mineiro*, *Cateto Roxo*, *Amarante*, *Gigante Lavinia*, *Moz 2014 Africa*, *Ito*, *San Valentin*, and *Chonan*. All of them are planted commercially in Brazil and are classified in three main groups (early, medium, and late planting) according to their climate requirements for bulbification. Temperatures around 20 °C, below 15 °C, and below 10 °C are required for proper bulbification of early (*Branco Mineiro,* and *Cateto Roxo*), medium (*Amarante*, *Gigante Lavinia*, and *Moz 2014 Africa*), and late (*Ito*, *San Valentin*, and *Chonan*) garlic cultivars, respectively. In addition, all these plants displayed yellowish mosaic in their leaves during vegetative development.

### RNA extraction, sequencing, and RT-PCR detection

Total RNA was extracted from symptomatic leaves of 10 plants from each garlic cultivar using the RNeasy^®^ Mini kit (Qiagen, Hilden, Germany) according to the manufacturer’s instructions. For high-throughput sequencing, the RNA samples were combined together (RNA pool). cDNA libraries and sequencing (2 × 100 bp read length) on the HiSeq 2,000 platform were performed at Macrogen Inc. (Seoul, Republic of Korea). The generated reads were trimmed and *de novo* assembled using CLC Genome Workbench 6.5.2 (CLC bio, Qiagen). Contigs related to viruses were retrieved using Blastx against a RefSeq virus database. To determine whether the assembled contigs corresponded to complete virus genomes, they were compared with complete virus genomes deposited on public databases using Geneious 7.1.8 (Kearse et al., 2012). Genome annotation was also performed using the latter program, in which open reading frames (ORFs) were annotated using BLASTx search against the NCBI non-redundant protein database.

The identified viruses were then traced back in each garlic cultivar by reverse transcriptase (RT) reaction followed by polymerase chain reaction (PCR) amplification. Complementary DNA sequences (cDNAs) were synthesized using SuperScript III reverse transcriptase (Thermo Fisher Scientific, Waltham, MA, USA) and random hexanucleotides. Then, PCR reactions were performed using PCR Master Mix (Promega, Madison, USA) and specific primer pairs for each one of the detected viruses (Table 1). Nucleotide (nt) sequences of PCR products were confirmed by Sanger sequencing at Macrogen Inc. All procedures followed the manufacturer’s instructions.

**Table 1:** Oligonucleotides used for the amplification of viral sequences.

**Virus**	**Oligonucleotides (5′ to 3′)** **forward (F)/reverse (R)**	**Oligos target ORF**	**Size of amplified fragment (bp)**
Garlic virus A	**F-**ACATCTTATGCCGCCCTTCT **R-**GGTGCGCTAGTCTCATCGTT	Replicase	*482*
Garlic virus B	**F-**ACTTCGCCTACATGCCTGG **R-**CCTGTGATTGACGGTGTGGT	Replicase	*700*
Garlic virus C	**F-**CATTTGCGGCGAACAATGGT **R-**TTGAGTTTTTGTTCTCTTGAGTTTGTG	Replicase	*671*
Garlic virus D	**F-**TTCCCAGCCTCTTCCCGG **R-**ACTTTCATCGTCACTCCAGTC	Replicase	*1,064*
Garlic virus X	**F-**GCCAGAGTTCGCGAGTTCTT **R-**CAAAGGTAGTTGACACGCTTGA	Replicase	*900*
Garlic common latent virus	**F-**GCATAGTACTTTCTGTCACC **R-**TATGCTTCATCCAGAGCTTT	Replicase	*975*
Garlic latent virus	**F-**TGAAGATTTGGAGGTGGGTTT **R-**CGGGTAATAAGCAACGGAGA	Replicase	*1,316*
Onion yellow dwarf virus	**F-**TCTTTAGTGACGATGCTTTTAAG **R-**AGATTTCAGATGCGATTTCACT	Polyprotein ORF (p1 protein /HC-Pro protein)	*1,170*
Leek yellow stripe virus	**F-**TGTAGTGGTGCGTTTCAGACA **R-**TGCTTTCCAATTCGCCCAATG	P1 protein	*731*
Garlic yellow mosaic-associated virus	**F-**GTGTGGCTAGTCTGCTTGGT **R-**TTGTGCTTGATCGCGGTTTC	Replicase	*1,000*

### Phylogenetic analysis

The phylogenetic tree containing ICTV recognized species of the family *Betaflexiviridae* was built based on the deduced amino acid (aa) sequences of the replicase and coat protein (CP) genes. For the cophylogeny trees, aa sequences of both replicase and CP were used. Multiple alignments were performed using the MAFFT method (Katoh & Standley, 2013). Then, maximum likelihood (ML) trees were inferred using PhyML (Guindon et al., 2010) under the JTT substitution model (Jones, Taylor & Thornton, 1992). Branch support was estimated by the Shimodaira-Hasegawa-like test (Anisimova et al., 2011). Cophylogeny analysis between the betaflexivirus trees was performed using the R program (R Core Team, 2013) with the Plytools (Schliep, 2018) and Phangom packages (Schliep, 2018). Finally, pairwise identity matrices were obtained using the SDT program (Muhire, Varsani & Martin, 2014) and plotted using Evolview (He et al., 2016).

## Results

The analysis of HTS data revealed the presence of viruses classified within genera *Allexivirus* (GVA, GVB, GVC, GVD, and GVX), *Carlavirus* (GCLV and GLV) and *Potyvirus* (OYDV and LYSV). Surprisingly, a new virus genome sequence which had close relationship to viruses of the family *Betaflexiviridae* was also found (Table 2). Each of these viruses was traced back in each garlic plant (cultivar) by RT-PCR. The betaflexivirus-like virus was detected in seven out of eight garlic cultivars. GVA and GVB isolates were the most frequent viruses, detected in all plants, while GVC and OYDV isolates were only detected in cv. *Gigante Lavinia* (Table 2).

**Table 2:** RT-PCR detection of garlic viruses in different Brazilian cultivars. Presence (+) or absence (−) of different garlic viruses are indicated.

Virus	Cultivar							
	*Branco Mineiro*	*Gigante Livinia*	*Amarante*	*Ito*	*San Valentin*	*Cateto Roxo*	*Chonan*	*Moz 214 Africa*
Garlic virus A	+	+	+	+	+	+	+	+
Garlic virus B	+	+	+	+	+	+	+	+
Garlic virus C	−	+	−	−	−	−	−	−
Garlic virus D	+	+	+	+	−	+	+	−
Garlic virus X	+	+	+	+	+	+	+	+
Garlic common latent virus	+	+	+	+	+	+	+	+
Garlic latent virus	+	+	−	−	−	−	−	+
Onion yellow dwarf virus	−	+	−	−	−	−	−	−
Leek yellow stripe virus	+	+	−	−	−	+	−	+
Garlic yellow mosaic-associated virus	+	+	+	+	+	+	−	+

The genome sequence of the putative new betaflexivirus was assembled from 3,881 reads. A reliable consensus sequence was obtained for this virus since a low number of mutations was observed after read mapping. Conversely, we could not achieve reliable complete genome sequences for the other viruses due to their high diversity and interspecific homology amongst themselves. Thus, only the complete genome sequence of the new putative betaflexivirus, tentatively named garlic yellow mosaic-associated virus (GYMaV), was deposited on the GenBank database under the accession number MH120170 (Fig. S1).

GYMaV has a positive sense, single-stranded RNA genome with 8,209 nt and five ORFs that encode a multi-domain replicase, the triple gene block proteins (TGB1, TGB2 and TGB3), and a CP (Table S1 and Fig. S1). The length and predicted molecular mass of each protein are displayed in Table S1. Since the CP and replicase gene sequences are the criteria for genus demarcation in the family *Betaflexiviridae* (King et al., 2012), a pairwise identity comparison was performed using all ICTV recognized species (75 sequences) (Fig. S2). GYMaV replicase shared 56% and 55% nt identity, respectively, with potato virus T (GenBank accession number EU835937, genus *Tepovirus*) and African oil palm ringspot virus (AY072921, genus *Robigovirus*). On the other hand, the GYMaV CP shares 64%, 62%, and 61% nt identity, respectively, with peach chlorotic mottle virus (EF693898), apple stem pitting virus (D21829), and apricot latent virus (HQ339956), all members of genus *Foveaviru*s. These values are well below the accepted species discrimination level of 72% nt identity for both CP and replicase (Adams et al., 2012). Even though the identity values were above the 45% nt identity threshold for genus demarcation, GYMaV should be considered a representative of the new genus of the family *Betaflexiviridae* as further discussed.

**Figure 1:**
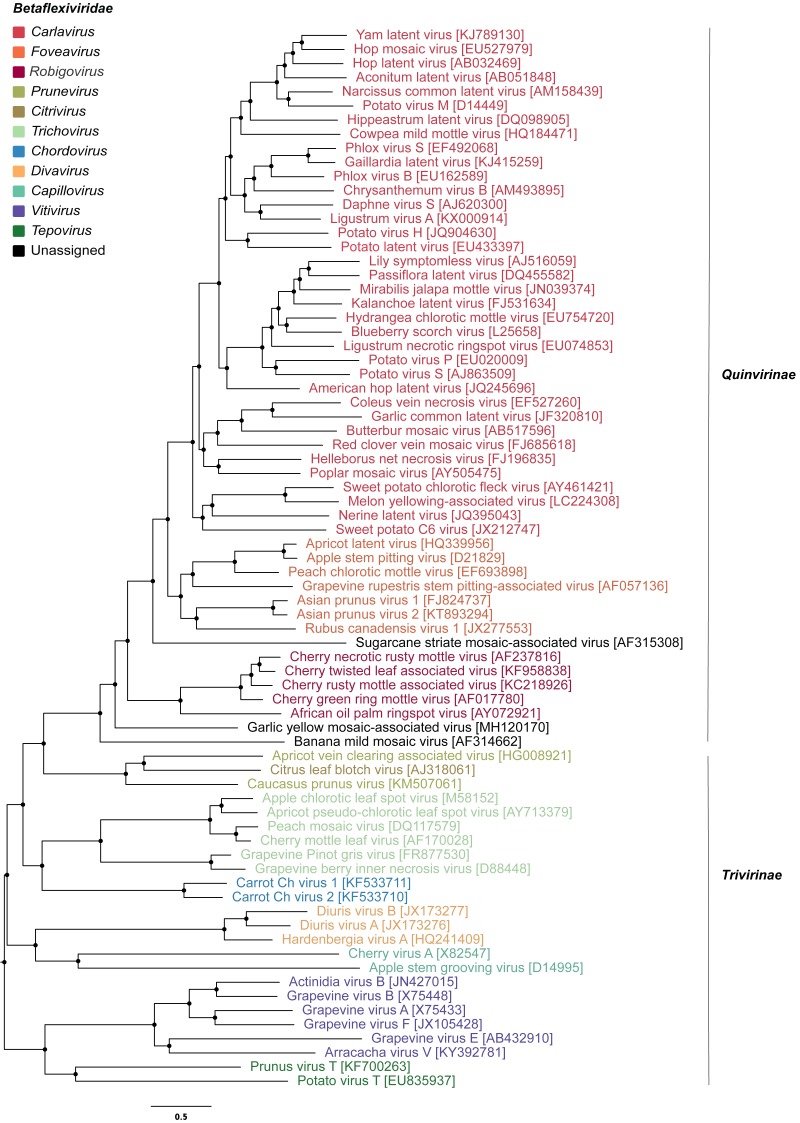
Phylogenetic tree of viruses classified within the family *Betaflexiviridae*. The taxa include representatives of different genera apart from viruses that are unassigned or unclassified as seen on NCBI. This tree was built on the replicase amino acid sequences of betaflexiviruses that have their ORFs completely sequenced.

**Figure 2:**
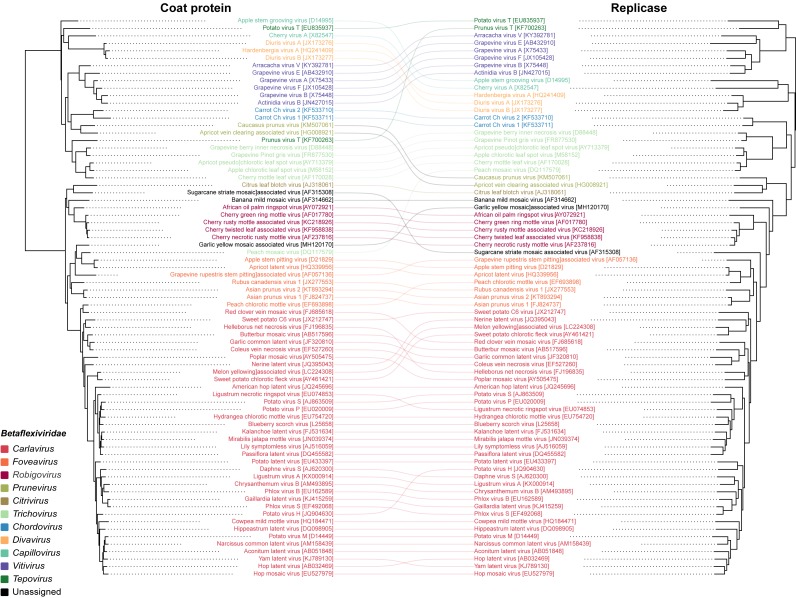
Cophylogeny of replicase and CP. The viruses listed include representatives of different genera of the family Betaflexiviridae. The colored lines indicate the taxa rearrangements for the replicase and CP

To infer the evolutionary relationships of GYMaV, a phylogenetic tree was constructed with replicase proteins (complete sequences) of ICTV recognized species in the family *Betaflexiviridae* (Fig. 1). Despite clustering with other viruses, GYMaV formed an independent and distant evolutionary lineage within this family. Since both the replicase and CP gene sequences are used for genus demarcation, a cophylogeny analysis was also performed. GYMaV clustered together with members of genus *Robigovirus* and the unassigned banana mild mosaic virus (AF314662) using replicase proteins. In contrast, GYMaV clustered within genus *Foveavirus* in CP phylogeny as suggested by pairwise comparisons (Figs. S2 and S3). Moreover, the trees were partially incongruent (Fig. 2), bringing up the question of whether these two viral genes should be considered for genus demarcation.

## Discussion

Aiming the identification of garlic-infecting RNA viruses following an unbiased approach, total RNA from eight garlic cultivars was high-throughput sequenced. Overall, virus isolates taxonomically classified in ten virus species were identified, nine of them having been previously reported in garlic VCs (Bereda, Paduch-Cichal & Dabrowska, 2017; Mituti et al., 2015; Wylie et al., 2014). The biological effects of these virus infections on the different garlic cultivars remains to be investigated, but based on previous studies they might compromise the growing of plants and bulbs (Conci, Canavelli & Lunello, 2003; Lunello, Rienzo & Conci, 2007). Although all plants presented yellow mosaic, it is hard to conclude whether all identified viruses are associated with this symptom or if there is a synergistic effect among the viruses in the community. In future research, this issue could be addressed via biological isolation of these viruses by mechanical or vector-borne inoculation/transmission onto indicator plants or by construction of infectious cDNA clones.

Apart from the viruses previously reported in garlic viromes, a new betaflexivirus, tentatively named garlic yellow mosaic-associated virus (GYMaV), was found in seven out of eight garlic cultivars tested. The presence of GYMaV in most cultivars indicates that it has likely been spread by vegetative propagation. However, its transmission by an insect vector should be not ruled out. Currently, the family *Betaflexiviridae* encompasses two subfamilies (*Trivirinae* and *Quinvirinae*) that together include eleven genera (https://talk.ictvonline.org/taxonomy/). GYMaV shares the highest nt identities with African oil palm ringspot virus (genus *Robigovirus*) and potato virus T (genus *Tepovirus*) for the replicase (56% and 55%, respectively) and with viruses classified in the genus *Foveavirus* for the CP (61–64% nt identity). According to the ICTV, viruses of suggested new genera are supposed to be less than 45% nt identical in those genes with viruses already reported (King et al., 2012). However, GYMaV constitutes a distant evolutionary lineage in the *Betaflexviridae* (Fig. 1), and therefore should be classified in a new genus. As seen in our pairwise identity matrices of theses genes, the sequence identity cut off should be revised since most comparisons for GaYMV are above 45% threshold (Fig. S2).

GYMaV virions may be shaped as flexuous filaments as observed for other betaflexiviruses (King et al., 2012). With a typical betaflexivirus genomic organization, GYMaV codes for three proteins (TGB1, TGB2 and TGB3) likely associated with cell-to-cell and systemic virus movement in plant hosts (Erhardt et al., 2005; Morozov & Solovyev, 2003). In general, betaflexiviruses have one (30K-like) or three movement proteins (as GYMaV), which is a criterion to assign them to subfamilies *Trivirinae* or *Quinvirinae*, respectively. Although the replicase and CP genes are used for genus demarcation, our analyses reinforce the concept of modular evolution, showing that these genes and protein products are phylogenetically incongruent. Thus, either one or another should be used for taxonomical purpose. Our analyses also suggest that either GYMaV underwent recombination or that these genes have different mutation rates due to different selection pressures.

GYMaV as a component of garlic VCs should be considered in the development of virus-free garlic varieties. Many surveys of garlic viruses previously reported were based on target specific methods, since specific detection tools were utilized (Chen & Adams, 2001; Chen, Chen & Adams, 2002; Fajardo et al., 2001; Fayad-Andre, Dusi & Resende, 2011; Nam et al., 2015; Taglienti et al., 2018). Although this is the first report of GYMaV, we cannot rule out its presence on a larger geographical and temporal scale.

## Conclusions

GYMaV is a putative new betaflexivirus found in virus complexes of several garlic cultivars. Based on its high frequency in these plants, GYMaV is likely to be vegetative propagated like other viruses previously reported in such complexes. Although the replicase and CP genes are used as taxonomical criteria for genus demarcation of the family *Betaflexiviridae*, cophylogeny analysis pointed that these genes sort out the betaflexiviruses differently.

##  Supplemental Information

10.7717/peerj.6285/supp-1 Figure S1 Genomic organization of and nucleotide sequence of GYMaV Abbreviations: triple gene block protein (TGB), Movement protein (MP), and Coat protein (CP).

10.7717/peerj.6285/supp-2 Figure S2 Pairwise identity aligments of *CP* and *replicase* genes/proteins from betaflexiviruses The numbers indicate the percentage of identical nucleotides or amino acids upon pairwise alignment.

10.7717/peerj.6285/supp-3 Figure S3 CP phylogenetic tree of viruses classified within the family *Betaflexiviridae* The taxa included CTV recognized species of different genera apart from viruses that are unassigned or unclassified as seen on NCBI. This tree was built on the CP predicted amino acid sequences of betaflexiviruses that have their ORFs completely sequenced.

10.7717/peerj.6285/supp-4 Supplemental Information 1 Proof of submission of a taxonomic proposal of new plant virus sequence to ICTV E-mail messages regarding submission of a taxoomic proposal of new plant virus genome sequence to ICTV.

## Additional Information and Declarations

### DNA Deposition

The following information was supplied regarding the deposition of DNA sequences: Garlic yellow mosaic-associated virus (GYMaV) genome sequence was deposited into the Genbank accession number MH120170. The sequence is also available as a Supplemental File.

### Data Availability

The following information was supplied regarding data availability: The genome sequence of the new virus is available as Figure S1.

### New Species Registration

The following information was supplied regarding the registration of a newly described species: This proposed new species [Garlic yellow mosaic-associated virus (GYMaV)] has been submitted to the ICTV for consideration. This name is only valid and official after the ICTV has approved the name, and it has been ratified by the membership.

## References

[ref-1] Adams MJ, Candresse T, Hammond J, Kreuze JF, Martelli GP, Namba S, Pearson MN, Ryu KH, Saldarelli P, Oshikawa N, King AMQ, Adams MJ, Carstens EB, Lefkowitz EJ (2012). Family *Betaflexiviridae*. Virus taxonomy: ninth report of the International Committee on Taxonomy of Viruses.

[ref-2] Anisimova M, Gil M, Dufayard JF, Dessimoz C, Gascuel O (2011). Survey of branch support methods demonstrates accuracy, power, and robustness of fast likelihood-based approximation schemes. Systematic Biology.

[ref-3] Ardisson-Araújo DMP, Rocha JR, Costa MHO, Bocca AL, Dusi AN, Resende RO, Ribeiro BM (2013). A baculovirus-mediated strategy for full-length plant virus coat protein expression and purification. Virology Journal.

[ref-4] Bag S, Schwartz HF, Cramer CS, Havey MJ, Pappu HR (2015). *Iris yellow spot virus* (*Tospovirus: Bunyaviridae*): from obscurity to research priority. Molecular Plant Pathology.

[ref-5] Bereda M, Paduch-Cichal E, Dabrowska E (2017). Occurrence and phylogenetic analysis of allexiviruses identified on garlic from China, Spain and Poland commercially available on the polish retail market. European Journal of Plant Pathology.

[ref-6] Camargo-Filho WP, Camargo FP (2015). A quick review of the production and commercialization of the main vegetables in Brazil and the world from 1970 to 2015. Horticultura Brasileira.

[ref-7] Chen J, Adams MJ (2001). Molecular characterisation of a complex mixture of viruses in garlic with mosaic symptoms in China. Archives of Virology.

[ref-8] Chen J, Chen JP, Adams MJ (2002). Characterisation of some carla- and potyviruses from bulb crops in China. Brief report. Archives of Virology.

[ref-9] Conci VC, Canavelli A, Lunello P (2003). Yield losses associated with virus-infected garlic plants during five successive years. Plant Disease.

[ref-10] Erhardt M, Vetter G, Gilmer D, Bouzoubaa S, Richards K, Jonard G, Guilley H (2005). Subcellular localization of the Triple Gene Block movement proteins of *Beet necrotic yellow vein virus* by electron microscopy. Virology.

[ref-11] Fajardo TVM, Nishijima M, Buso JA, Torres AC, Avila A, Resende RO (2001). Garlic viral complex: identification of potyviruses and carlavirus in central Brazil. Fitopatologia Brasileira.

[ref-12] Fayad-Andre M, Dusi AN, Resende RO (2011). Spread of viruses in garlic fields cultivated under different agricultural production systems in Brazil. Tropical Plant Pathology.

[ref-13] Guindon S, Dufayard JF, Lefort V, Anisimova M, Hordijk W, Gascuel O (2010). New algorithms and methods to estimate maximum-likelihood phylogenies: assessing the performance of PhyML 3.0. Systematic Biology.

[ref-14] He Z, Zhang H, Gao S, Lercher MJ, Chen W, Hu S (2016). Evolview v2: an online visualization and management tool for customized and annotated phylogenetic trees. Nucleic Acids Research.

[ref-15] Jones DT, Taylor WR, Thornton JM (1992). The rapid generation of mutation data matrices from protein sequences. Bioinformatics.

[ref-16] Kang SG, Koo BJ, Lee ET, Chang MU (2007). Allexivirus transmitted by eriophyid mites in garlic plants. Journal of Microbiology and Biotechnology.

[ref-17] Katoh K, Standley DM (2013). MAFFT multiple sequence alignment software version 7: improvements in performance and usability. Molecular Biology and Evolution.

[ref-18] Kearse M, Moir R, Wilson A, Stones-Havas S, Cheung M, Sturrock S, Buxton S, Cooper A, Markowitz S, Duran C, Thierer T, Ashton B, Meintjes P, Drummond A (2012). Geneious Basic: an integrated and extendable desktop software platform for the organization and analysis of sequence data. Bioinformatics.

[ref-19] King AMQ, Adams MJ, Carstens EB, Lefkowitz EJ (2012). Order—tymovirales, virus taxonomy.

[ref-20] Lunello P, Di Rienzo J, Conci VC (2007). Yield loss in garlic caused by *leek yellow stripe virus* Argentinean isolate. Plant Disease.

[ref-21] Mituti T, Moura MF, Marubayashi JM, Oliveira ML, Imaizumi VM, Sakate RK, Pavan MA (2015). Survey of viruses belonging to different genera and species in noble garlic in Brazil. Scientia Agricola.

[ref-22] Morozov SY, Solovyev AG (2003). Triple gene block: modular design of a multifunctional machine for plant virus movement. Journal of General Virology.

[ref-23] Muhire BM, Varsani A, Martin DP (2014). SDT: a virus classification tool based on pairwise sequence alignment and identity calculation. PLOS ONE.

[ref-24] Nam M, Lee YH, Park CY, Lee MA, Bae YS, Lim S, Lee JH, Moon JS, Lee SH (2015). Development of multiplex rt-PCR for simultaneous detection of garlic viruses and the incidence of garlic viral disease in garlic genetic resources. Plant Pathology Journal.

[ref-25] R Core Team (2013). https://www.R-project.org/.

[ref-26] Schliep KP (2018). https://cran.r-project.org/web/packages/phangorn/phangorn.pdf.

[ref-27] Taglienti A, Tiberini A, Manglli A, Rea R, Paoletti S, Taviani P, Tomassoli L (2018). Molecular identification of allexiviruses in a complex mixture of garlic viruses in Latium (central Italy). European Journal of Plant Pathology.

[ref-28] Tsuneyoshi T, Matsumi T, Deng T, Sako I, Sumi S (1998). Differentiation of Allium carlaviruses isolated from different parts of the world based on the viral coat protein sequence. Archives of Virology.

[ref-29] Turina M, Kormelink R, Resende RO (2016). Resistance to tospoviruses in vegetable crops: epidemiological and molecular aspects. Annual Review of Phytopathology.

[ref-30] Wylie SJ, Li H, Saqib M, Jones MG (2014). The global trade in fresh produce and the vagility of plant viruses: a case study in garlic. PLOS ONE.

