# Supplementary material for: A new virus found in garlic virus complex is a member of possible novel genus of the family Betaflexiviridae (order Tymovirales)
Source: PeerJ. 2019 Jan 16;7:e6285. doi: 10.7717/peerj.6285 (PMC6339470; doi:10.7717/peerj.6285)
Supplement: Figure S1 — Abbreviations: triple gene block protein (TGB), Movement protein (MP), and Coat protein (CP). [file peerj-07-6285-s001.pdf]

Garlic yellow mosaic-associated virus

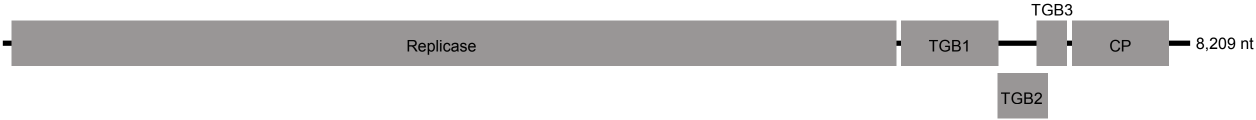

```
LOCUS       Garlic                  8209 bp    RNA     linear
23-MAR-2018
DEFINITION  yellow mosaic-associated virus.
ACCESSION   Garlic
VERSION
KEYWORDS    .
SOURCE      Garlic yellow mosaic-associated virus
  ORGANISM  Garlic yellow mosaic-associated virus
            Unclassified.
REFERENCE   1  (bases 1 to 8209)
  AUTHORS   da Silva,L.A.
  TITLE     A member of a new virus genus from family
Betaflexiviridae (order
            Tymovirales) being part of the virome of garlic (Allium
sativum)
            samples
  JOURNAL   Unpublished
REFERENCE   2  (bases 1 to 8209)
  AUTHORS   da Silva,L.A.
  TITLE     Direct Submission
  JOURNAL   Submitted (23-MAR-2018) Celular Biology, University of
Brasilia,
            Campus Universitario Darcy Ribeiro, Brasilia, Distrito
Federal
            70790150, Brasil
COMMENT     Bankit Comment: ALT EMAIL:leocbq@yahoo.com.br.
            Bankit Comment: TOTAL # OF SEQS:1.

            ##Assembly-Data-START##
            Assembly Method       :: Geneious v. 10.0.2
            Sequencing Technology :: Illumina
            ##Assembly-Data-END##
FEATURES             Location/Qualifiers
     source          1..8209
                     /organism="Garlic yellow mosaic-associated
virus"
                     /mol_type="other RNA"
                     /host="Allium sativum"
                     /country="Brazil"
     CDS             53..6178
                     /note="229.9 kDa; ORF1"
                     /codon_start=1
                     /product="replicase"
                     /
translation="MAFSYKTPVESLLSKFTSDEQSKICSTAAEKIASLEKAEHNFFS

YYLSDEAKKILTERGVRLSVVAHQVHSHPVCKTVENHILFNVIKSYIDNSFYVVSIKS

KKLEYLRARNKMPTLSYINRYIAAKDISRYGMDKYFNEIAESEVTGILNRKKKRFFNL

FESAKKEFNSANYDPSNSETLKDLVPQALTTKAKRFFLHDELHYWSLEELDLFLCTVK

PEIIVATHVFPKEILKGVHHSYNKWMYDFKVEGEKLFYYPDGCLEEGYEQPMHGGLMY
```

YLNSFTTTTGEIYSISLVYNLFSHSVFQISKGNLKTETQRSFSGFQASPTNELTAFGG

TILKTFGVKSSLIKKIYLYLRTLKKPDIESAMAKLRQLEPDPSGQTIVFVENFSEFLL

SHKTGRNLIEEGLASKFEKFIIGLLPNLVRKNFESHYSSNLCEFISNLEDDKVVVNCQ

CGDSKFSDWSVVYPKIDEMRRKLINEVVKFKDPVMELFSNHLKDGKLRRRPQKYFISN

AGTEMLGESRTTMAKKAAQFFLYSFSCSGLKFAKKRILNEVMRNDFTFFMRNISFRRC

VEAGNIDHISFLIFLRNYFDERFRKISDDPLLLTYRSCSEEEEKKKAIKQAIEESKRK

FSGVIKELFNDNKFKIKGIISDLYTVESFRSQDPEPTHLNSVEQFQSEFDSLVLPAVE

GAVSSTEFDEKKGDCKSLIDISDEIVPQDPFAAFFLSGSINSSVEMAQPLEPTVYSSH

YHYCGCGLKIVVNSFNNEIAISGMIFCDSMKGRMGAFYSRDNSGYAYKGFSHSSQGWL

NGLDKLISACGEEPTDYNQCLAQRYEEGSGIGFHSDDEAIYPKGNKILTVNASGSGQF

GIRCNADEFYLNLNDGDFFIMPCGFQESHKHRVTALSVRVSLTFRSTVANCCEKKNEL

VPENLRSDCIESSVQNLSLPNSFYRVKLNSIHTLSQRFIISDIDKFNEFKVVGDGNCF

WHCVASLLGGSAETCKEICKNHCEAHHLAPSEKLQFDGSNWAEDESIFIACTAFSIRI

TMFDLVSSVVHEFKPVSSNEELNVNIVFDGSHFNLLVPKEGCVIRAIAESLNRKEIEI

VKVLALNENSKILEELNTGLGLPMNLLEDCFKIFGIRALVNFGTESIEFNKQGKLCRK

FFLTDGHIEYIGLANFDSKLGQKKISLSNKNYDSIFASMADGNVISYVPSLDRAKLLE

KSFKDGLTGKILATSFGGPEMIIKKDVEAIKKKVWFMVGTFGSGKSFSVKNKIKERTD

INFLIISPRRKLADVFKEELGLKKEWKKAKKSNFEVVTFETAIKHKSFKKSEIIILDE

LQLFPPGYLDLLLLISKSENILVLGDPAQSSYDSEEDRAIFEGINNDLVNLLSNQKYN

YLIQSKRFRNRFIDGRLPCKFDDLSNFPSEEYFVYEDMRKESDLILTNDVILCSSFDE

KKSISYILGRRREVLTFGESTGLTFRKVCIVLTQNFRMTDEKRILVALSRASYQTNFV

NNTGLPFKDFILSMPNSVIFKYCSATCNVSDLLPLLPGEPNFISTRVRIGHDEVDREA

RMIGDPWLKTMLFLGQRGSYEENVPFEEPVLEMRTLTHHPIVGDNVVRARISELFRSK

EEREFRIDDNVSEQFRDSYNVKDFFKSSNQCELFEAIYPRHKGTDVVTFLMAVRKRLS

FSDPAVNESKFNSAKTFGLLMFEHFVKYIPLKSNRDEEMFETARSDFERKKLEKNIAT

IENHSGRSSADWDIREAFVFMKSQLCTKFEKRFVDAKAGQTLACFSHIVLCRFAPWIR

YIEKKVFEVLPPNFYIHSGKNFDELKEWVLRSDFSGECTESDYEAFDASQDATILSFE

```
VEIMKYLNIPHDVIEDYKFIKFNLFSKLGIFEIMRFTGEAGTFLFNTLANICFTLMRY

KIRGDECIAFAGDDMCANTCLRVSTEFENILDRLKLKAKVDYKSQASFCGWSLGPYGI

YKKPQLVFERFMISKEKGTLHECIDNYAIEVSYGYRMGDRVFGYMTEEEIECQNLCIR
                     TIVLNKQMMKETALSYFNGSLSRLE"
     CDS             6209..6883
                     /note="25.0 kDa; ORF2"
                     /codon_start=1
                     /product="triple gene block protein 1"
                     /
translation="MNKLFELLTEFEFVRTNIPISRPLVVHAVPGAGKTTLLRKFLNT

CSSAEVITSGVGDKPNLLGKRIVHGNQFSIEGSFRIFDEYITSELIPDCEALFSDPIQ

NNKEGLPAHYIKKKSLRVPKAICDWLQTLGFEIESEVEGELSFQNFFGPDPVGKIVAF

ETNVLELLRRHNCDFSLPCEIRGLEFNTVTLFTERDCSVLSGFELYIAATRARKKLIV
                     RTADAN"
     CDS             6873..7217
                     /note="13.0 kDa; ORF3"
                     /codon_start=1
                     /product="triple gene block protein 2"
                     /
translation="MPISAPPDYSKTFVIIAVGAGIALCLFILTRSTLPSVGDNIHQL

PHGGTYVDGTKRINYCGPNKEFPSSNLFNPGSNFGVLLLVITLIFAIHVLSGRETTIR
                     SNCGCIHHSSRN"
     CDS             7147..7356
                     /note="7.1 kDa; ORF4"
                     /codon_start=1
                     /product="triple gene block protein 3"
                     /
translation="MFSQDVKLLFGVIVVAFIILHVIDLQRQEACTILIDGSKVLIQS
                     CNLSPEVIEKLAKLKPMNHGLSLNR"
     CDS             7393..8061
                     /note="29.7 kDa; ORF5"
                     /codon_start=1
                     /product="coat protein"
                     /
translation="MNKDIQEKGASPLVGKVKENKISMKEFENLEIGFETNKVATQMQ

INEIKARFLELGIPDEKAALAFVDIALQCADMGSSDQTKLVGNSAVNVKVRRESLVAV

IKNTCSLRQFCAYYAKIVWNLLLSHNRPPANWHSKGFRDSEKYAAFDFFFGVDHESSI

NPAEGLYRKPTEKERVANESSKEVSIYRQIYREGNNVLNLGEVTGGKAGYKASLNFGK
                     SQTE"
     3'utr           8066..8209
BASE COUNT     2631 a   1434 c   1729 g   2415 t
ORIGIN
        1 aacgactgta gacgacacaa ctacaaattg tctcccctct acactctcta
taatggcctt
       61 ctcatacaag acccctgttg aatctctact gtcaaagttc acttctgatg
```

aacaatctaa
121 aatttgcagc actgcagctg aaaaaattgc atcattggaa aaagccgaac acaacttctt
181 ctcttactac ttaagtgatg aagcaaagaa aattctcacc gaaagaggcg taagattatc
241 tgttgtggct caccaagtgc atagccatcc agtatgtaaa acagtagaga accacatttt
301 gtttaatgta attaaaagtt atatagataa ttctttttat gtagttagca ttaagagtaa
361 aaagttagag taccttaggg caagaaataa gatgcctacg ctcagttata tcaataggta
421 tatagctgct aaagatataa gcagatacgg catggacaag tacttcaacg aaattgctga
481 aagtgaagta actggcattc tcaataggaa aaagaaaaga ttctttaact tatttgagag
541 tgcaaagaag gaattcaatt cagccaatta cgatccatcc aattcagaaa cacttaaaga
601 tttggtgcca caagctctca ccacaaaggc taagagattt ttcttgcatg atgaattaca
661 ttattggagt cttgaggaac tagatctttt tctatgcaca gtcaaaccgg aaatcattgt
721 tgctacacat gtttttccaa aagaaatttt gaaaggtgtg catcacagtt acaacaaatg
781 gatgtatgac ttcaaagttg aaggagaaaa attattttat tatccagatg gctgcctaga
841 agagggttat gagcaaccca tgcacggggg gcttatgtat tatttgaatt cctttacgac
901 taccaccggc gaaatttact caataagtct ggtttacaat ctcttttcac attcagtctt
961 ccaaatctct aagggtaact taaaaacgga gacacaaaga agctttagcg gcttccaggc
1021 atcaccaacc aacgagttaa ctgcctttgg aggaacgata ctcaaaactt ttggagtcaa
1081 aagttcctta atcaaaaaaa tttatcttta cttaaggacg ctaaagaaac cagacattga
1141 atccgcaatg gctaaactca ggcaacttga acctgaccct tctggtcaaa ctatagtttt
1201 tgttgaaaat ttctcggagt tcctactttc acacaaaaca ggtagaaatt taattgagga
1261 gggtttagca agcaaatttg aaaaattcat tattggtttg ttgcctaacc ttgtgcgtaa
1321 aaattttgag tcccactaca gctccaatct ttgtgagttt atctcaaatc ttgaagatga
1381 caaagtggtt gtgaattgtc agtgtggcga ttccaaattc tctgactgga gtgttgtgta
1441 tccaaagatt gatgaaatga gaagaaaatt aattaatgaa gttgtcaaat tcaaggatcc
1501 agttatggaa cttttctcaa atcatctcaa ggatggcaaa ttaaggagaa ggcctcaaaa
1561 atactttatt tctaatgctg gaacagaaat gctcggtgaa tctagaacaa ctatggcaaa
1621 gaaggctgct cagttcttcc tatatagttt ctcttgctct gggctcaaat ttgcaaagaa
1681 aagaattctg aatgaagtta tgcgtaatga tttcactttt ttcatgagaa

atatttcgtt
1741 tagaaggtgt gttgaggctg gaaatattga tcatatctca ttccttattt tcctacgaaa
1801 ctattttgat gagaggttca gaaaaattag tgatgatcct ctcttgctga cgtacagatc
1861 atgtagtgaa gaggaggaaa aaaaaaaagc tattaagcag gccatagaag aatccaaaag
1921 gaaattcagt ggggtgataa aagagctttt taatgacaat aaattcaaga ttaaaggtat
1981 tatttctgat ctgtacactg ttgaatcctt taggagtcag gatccagaac ccactcatct
2041 aaacagtgtt gagcagttcc aatctgagtt tgactcgttg gtcttacctg cagtggaggg
2101 agctgtttct tcaactgaat ttgatgaaaa aaagggggat tgtaaatcat tgatagacat
2161 cagcgatgaa atcgtgccac aagatccttt tgctgcattt tttttgagtg gatcaatcaa
2221 ctcctcagtt gagatggctc aacctcttga acctactgtt tattcctcac attatcatta
2281 ttgcggatgc gggttaaaga ttgttgtcaa ttctttcaat aatgagatag ctatcagcgg
2341 aatgattttc tgtgatagca tgaaagggcg gatgggtgcc ttttactcca gggataactc
2401 ggggtatgct tataaaggtt tttcgcattc atcacagggg tggcttaatg gcttagacaa
2461 attgatttct gcttgtggtg aggaaccgac tgattacaat caatgcttgg cccaacggta
2521 tgaagaagga tcaggtatag gattccatag tgatgacgaa gcaatttacc caaaagggaa
2581 taaaattttg accgtaaatg cttctgggtc tggtcaattt ggaatcagat gcaacgcaga
2641 tgagttctat ctaaatttga atgatgggga tttcttcata atgccttgtg ggttccaaga
2701 aagccataag catagagtga ctgctttgtc agtaagggtc tcgctcactt ttagatcaac
2761 agttgctaat tgctgtgaga agaaaaatga acttgtccct gagaatctga gaagtgactg
2821 catagagtca agcgtgcaga atttatcgct acccaattcc ttttaccgag tgaaattgaa
2881 ctcaattcac actttgagtc aaaggttcat tatttctgac attgataaat tcaatgaatt
2941 caaggtagtt ggagatggga attgcttctg gcattgtgtg gctagtctgc ttggtgggtc
3001 agctgaaact tgtaaagaaa tatgcaaaaa tcattgtgaa gcacatcatt tggcaccttc
3061 cgagaaattg caatttgatg gtagtaattg ggccgaggat gaatcgatct tcatcgcttg
3121 caccgctttc tcgattagaa taacgatgtt tgatttggtg tcctctgttg ttcatgaatt
3181 caagcctgtg tcttcaaatg aggagctgaa tgtgaatatt gtgtttgatg gctctcattt
3241 caatttacta gttccaaaag aaggctgtgt cattcgagca attgctgaat cattgaatag
3301 gaaagagatc gaaatcgtca aagtcctggc tcttaatgaa aattcaaaga

tactcgaaga
3361 acttaatact ggtttaggtc taccaatgaa tctgctggaa gattgcttta
aaatctttgg
3421 cattcgggcc ttggttaatt tcggaactga aagtatagaa ttcaacaagc
aaggaaaatt
3481 atgcagaaaa ttcttcctta ctgatggcca cattgagtat atcggactgg
ccaactttga
3541 ctctaaactt ggtcaaaaaa aaatatcatt gagcaataaa aattatgact
caatcttcgc
3601 aagcatggct gatggcaatg tgatcagtta cgtcccaagt cttgacaggg
cgaaactact
3661 tgagaaatca tttaaagatg gattaactgg taagattctc gccacaagct
tcggtggccc
3721 ggagatgatc atcaagaaag atgttgaggc cataaagaag aaggtttggt
tcatggttgg
3781 cactttcggt tcaggaaagt cctttagcgt taaaaataaa ataaaagagc
gaactgatat
3841 caattttctc atcatttccc caagaaggaa gctcgcagat gtctttaagg
aagaattggg
3901 tttgaagaag gagtggaaaa aagcaaagaa aagcaatttt gaagttgtga
ctttcgaaac
3961 cgcgatcaag cacaaaagct tcaagaaaag tgaaattatc atccttgatg
agttacagtt
4021 gtttccgcca gggtacttgg acttgttgct tctgataagc aagtctgaaa
acatactggt
4081 gcttggtgat cccgcccagt cgagctatga cagtgaagag gatagagcaa
tttttgaagg
4141 tatcaataac gaccttgtca atctgctttc aaatcaaaag tacaactacc
taattcagtc
4201 aaaaaggttt agaaatagat tcattgacgg caggttacct tgcaaatttg
acgatctttc
4261 caactttcct tcagaagaat actttgtgta tgaagatatg aggaaagagt
cagacctcat
4321 tctgaccaac gatgttatac tttgcagttc atttgatgaa aaaaaaagca
tatcttacat
4381 acttgggagg agaagagagg ttttgacctt tggagagtca acaggtctga
cgtttagaaa
4441 ggtttgcatt gttttaactc agaatttcag aatgacagat gagaagcgaa
tccttgtagc
4501 attatccaga gcttcatatc aaactaattt tgtcaataac actgggctcc
cttttaagga
4561 tttcatacta tccatgccca attctgttat tttcaaatat tgctcagcca
cgtgcaacgt
4621 gtcagatctg cttccactgc taccaggtga acctaacttt atatccacac
gcgttagaat
4681 tggacatgac gaagtggata gagaagccag aatgattggg gatccatggc
ttaaaacaat
4741 gctttttta gggcagaggg gcagttatga agaaaatgtt ccgtttgaag
agcctgtgct
4801 tgaaatgaga accttgactc accatccaat agttggagat aatgtggtta
gggctagaat
4861 ttctgaacta tttcgttcaa aagaagagcg cgaattcagg attgacgata
atgtttcaga
4921 gcagtttcgt gactcttaca atgtaaagga tttctttaaa agttcaaatc

aatgtgaact
4981 ttttgaagca atatatccaa gacacaaagg gactgatgta gttacctttt taatggctgt
5041 aaggaagagg ttatcattct ctgacccagc tgtaaacgaa agtaaattca attctgctaa
5101 aacttttggg ctgttgatgt tcgagcattt tgtcaagtat atacctctta aaagcaatag
5161 ggacgaggaa atgtttgaaa ccgcaagatc tgatttcgaa agaaagaaac ttgaaaaaaa
5221 catagctacc attgagaatc attcaggcag atccagtgct gattgggata ttagagaggc
5281 atttgtcttc atgaagagcc aactatgcac aaaatttgaa aaaagatttg tggatgcaaa
5341 ggcaggacag acactggcct gcttcagtca tatagtccta tgccgatttg ctccatggat
5401 taggtatatt gaaaagaaag tctttgaagt gctccctcca aatttttaca tacattccgg
5461 gaaaaacttt gatgaactca aggaatgggt actcagatct gatttttccg gtgaatgtac
5521 cgaatctgat tatgaagctt ttgatgcctc tcaagatgca acgatcttga gttttgaggt
5581 tgagataatg aaatacttga atattcctca tgatgtaatt gaagactaca aatttatcaa
5641 attcaatctc ttcagcaaac taggaatctt cgaaataatg agattcactg gtgaagcagg
5701 tacatttcta ttcaacacac tcgcgaatat atgcttcact ttgatgcgat acaaaataag
5761 gggggatgaa tgtatcgcct ttgcaggtga tgatatgtgt gcaaacactt gtctaagggt
5821 ttcaactgag ttcgagaaca ttctagatag gctcaaactt aaagcaaaag ttgactacaa
5881 aagtcaggct tcattctgcg gttggtcact tggtccatat ggcatttata aaaagccaca
5941 attggtattt gaacgcttca tgatatccaa ggagaagggc accctgcacg aatgcataga
6001 caattatgca atagaggttt catatggcta taggatgggt gaccgagtct ttggatacat
6061 gactgaggaa gaaattgagt gccagaacct gtgcatcaga accatagtac tgaataaaca
6121 aatgatgaag gagacagccc ttagttactt taatgggtcg ttaagcaggt tagagtaaat
6181 agcttaggtt gtaactgtta ggtagataat gaataaatta tttgaattac taactgaatt
6241 tgaatttgtt agaactaata tacccatttc cagaccctta gttgttcacg ctgttcctgg
6301 agctggaaaa acaacattgt tgaggaagtt cctaaatacc tgctcctctg cagaggtaat
6361 cacatcagga gttggagaca aaccaaacct attgggtaaa agaattgttc acggcaacca
6421 atttagcatt gaaggaagct tcagaatctt tgacgaatac ataacttcag aattgatccc
6481 ggattgtgag gctcttttct ccgatccaat tcaaaataac aaagaagggc tacctgctca
6541 ttatatcaaa aagaaatctt taagggttcc taaagcaatc tgtgattggc

tgcaaacgct
6601 aggtttcgaa attgagtcag aagttgaagg tgaactctcc tttcaaaatt tctttggacc
6661 tgatccagtt ggtaaaatcg ttgcttttga aacaaacgtg ctagaactct tgaggagaca
6721 taactgtgat ttttctctac cttgcgaaat cagaggtttg gaattcaata ctgttacact
6781 cttcactgaa agggattgtt cagtgctgtc aggttttgaa ttgtacattg ctgccaccag
6841 agcaagaaag aagttaattg tgaggacagc agatgccaat tagtgcaccc cctgactact
6901 caaaaacttt cgtgatcatt gcagttggag ctggaattgc actttgcttg ttcatattaa
6961 ccaggtcgac attacccagt gttggagata acatacatca gttacctcac ggaggtacat
7021 acgtcgacgg aacaaaaaga atcaactact gcgggcctaa caaggaattc ccaagttcca
7081 accttttcaa tccagggtca aattttggtg ttttgctctt ggtgatcaca ttaatatttg
7141 caattcatgt tctctcagga cgtgaaacta ctattcggag taattgtggt tgcattcatc
7201 attcttcacg taattgacct ccaaagacag gaagcctgca caatattaat agacggctca
7261 aaggtcctga tccagagttg caatttatct cctgaagtca tagaaaagct tgccaagttg
7321 aagcctatga atcatggctt aagtcttaac cgttaaattg aaatagtaac aactctgatt
7381 taagagaaag acatgaacaa ggacattcaa gagaaaggtg caagtcctct ggttgggaaa
7441 gttaaagaaa ataaaatatc tatgaaggaa tttgagaatc tagaaatcgg ctttgagaca
7501 aacaaggttg ccacccagat gcagataaat gaaataaagg cccgatttct agaactcggg
7561 attccagatg aaaaagctgc tttggccttc gtagatattg cattgcaatg cgctgatatg
7621 ggcagttctg accaaacaaa gcttgttggg aactcagcgg tcaatgtgaa agttaggcgt
7681 gaaagcctag ttgctgtgat taaaaacact tgctcattga gacaattttg tgcttattat
7741 gccaaaatcg tttggaattt attgctgtct cacaacagac ctccagccaa ttggcattct
7801 aagggcttta gagatagtga gaaatatgct gccttcgatt tcttcttcgg tgtggatcat
7861 gaaagttcaa tcaatccagc tgaaggttta tacaggaaac caactgagaa agagagagtt
7921 gccaatgaat cttcgaaaga ggtgtcgatc tatagacaga tttacaggga gggcaataat
7981 gtcctcaacc ttggtgaagt tactgggggt aaggctgggt ataaagccag cttaaatttc
8041 ggaaaatcac agaccgaatg aattacattg agcttcaatg agttttccag attttgagtg
8101 gataaatctg gttgtgggat ttctatgagg tctttgctca caatattgta gacctcagtt
8161 tgccagtttt ctataaaata aaccgactta atagtgttgt aaatggctt

//
