# Supplementary figures and images for: A new virus found in garlic virus complex is a member of possible novel genus of the family Betaflexiviridae (order Tymovirales)
Source: PeerJ. 2019 Jan 16;7:e6285. doi: 10.7717/peerj.6285 (PMC6339470; doi:10.7717/peerj.6285)

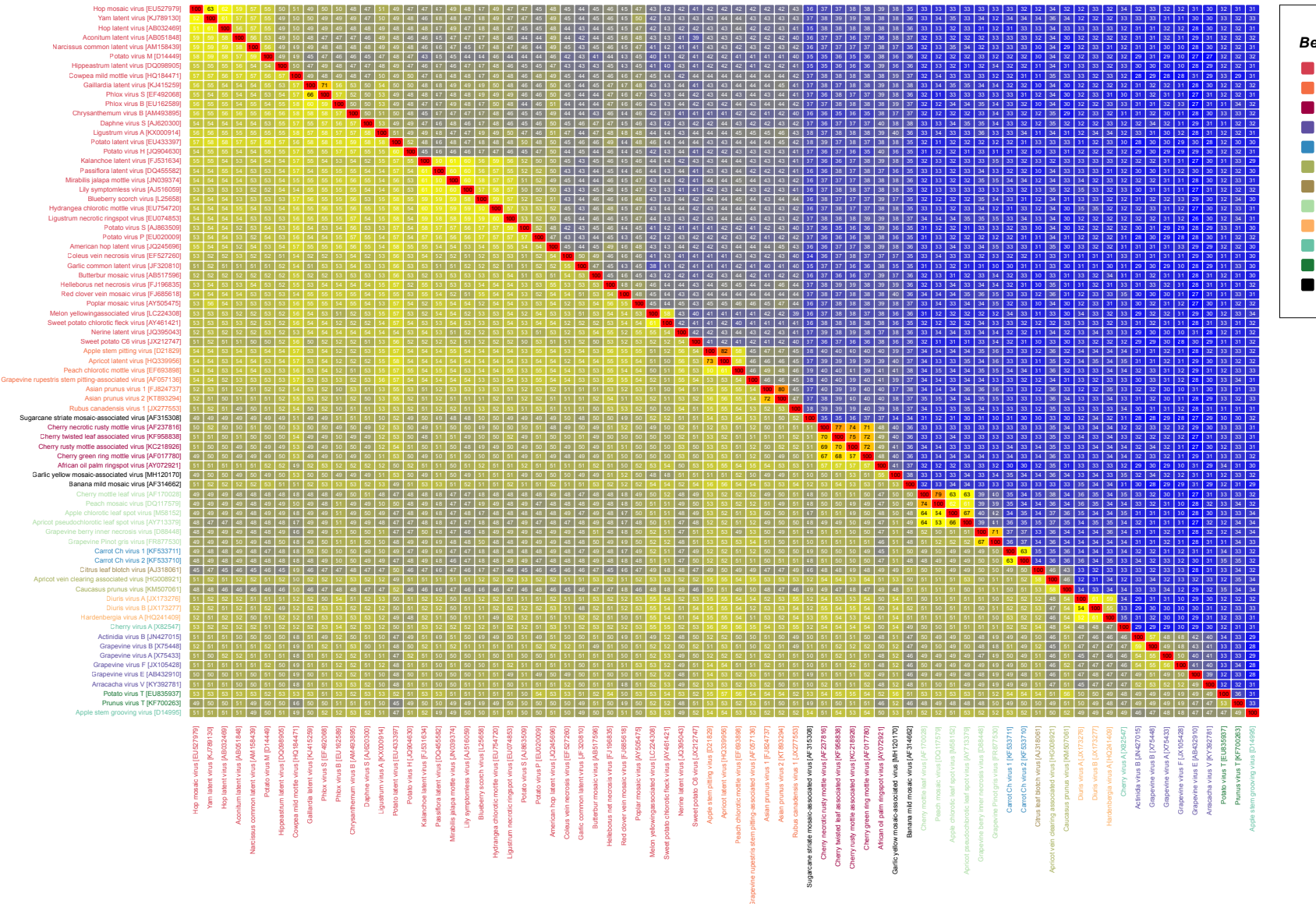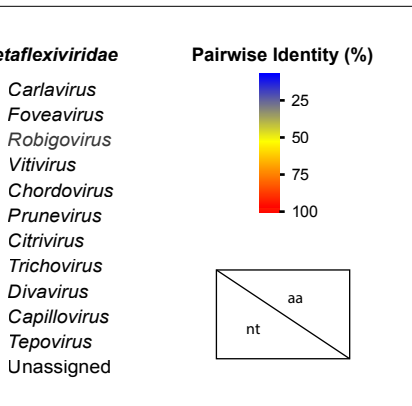

Replicase

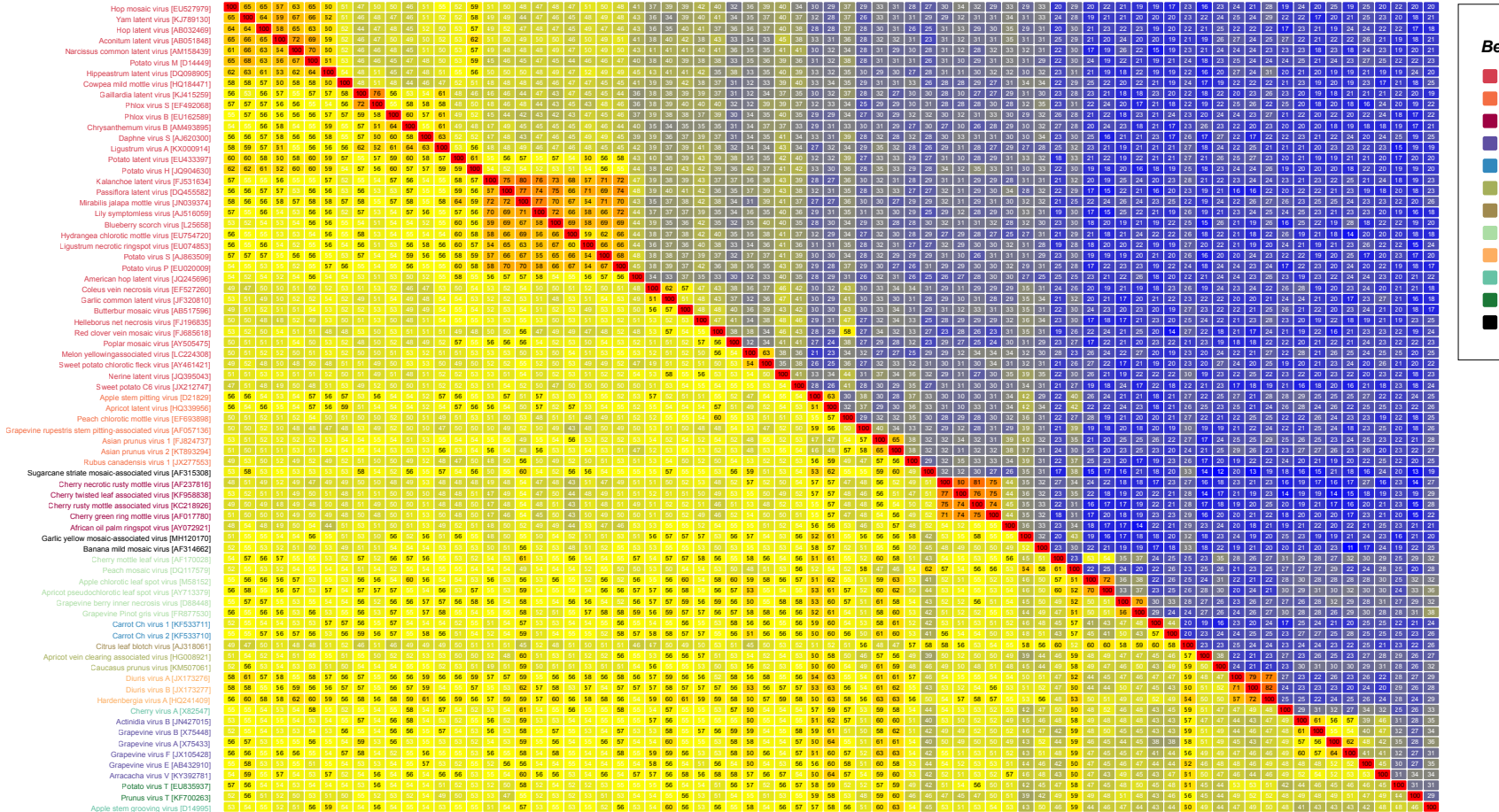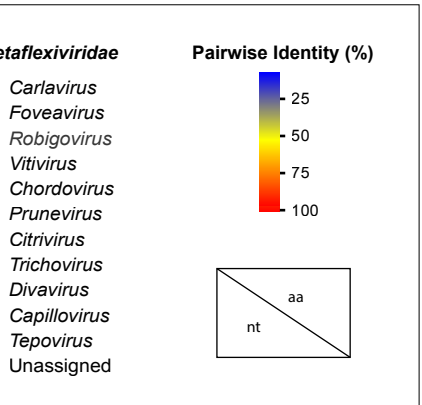

Coat protein

Supplement: Figure S2 — The numbers indicate the percentage of identical nucleotides or amino acids upon pairwise alignment. [file peerj-07-6285-s002.pdf]
