# Supplementary material for: A new virus found in garlic virus complex is a member of possible novel genus of the family Betaflexiviridae (order Tymovirales)
Source: PeerJ. 2019 Jan 16;7:e6285. doi: 10.7717/peerj.6285 (PMC6339470; doi:10.7717/peerj.6285)
Supplement: Figure S3 — The taxa included CTV recognized species of different genera apart from viruses that are unassigned or unclassified as seen on NCBI. This tree was built on the CP predicted amino acid sequences of betaflexiviruses that have their ORFs completely sequenced. [file peerj-07-6285-s003.pdf]

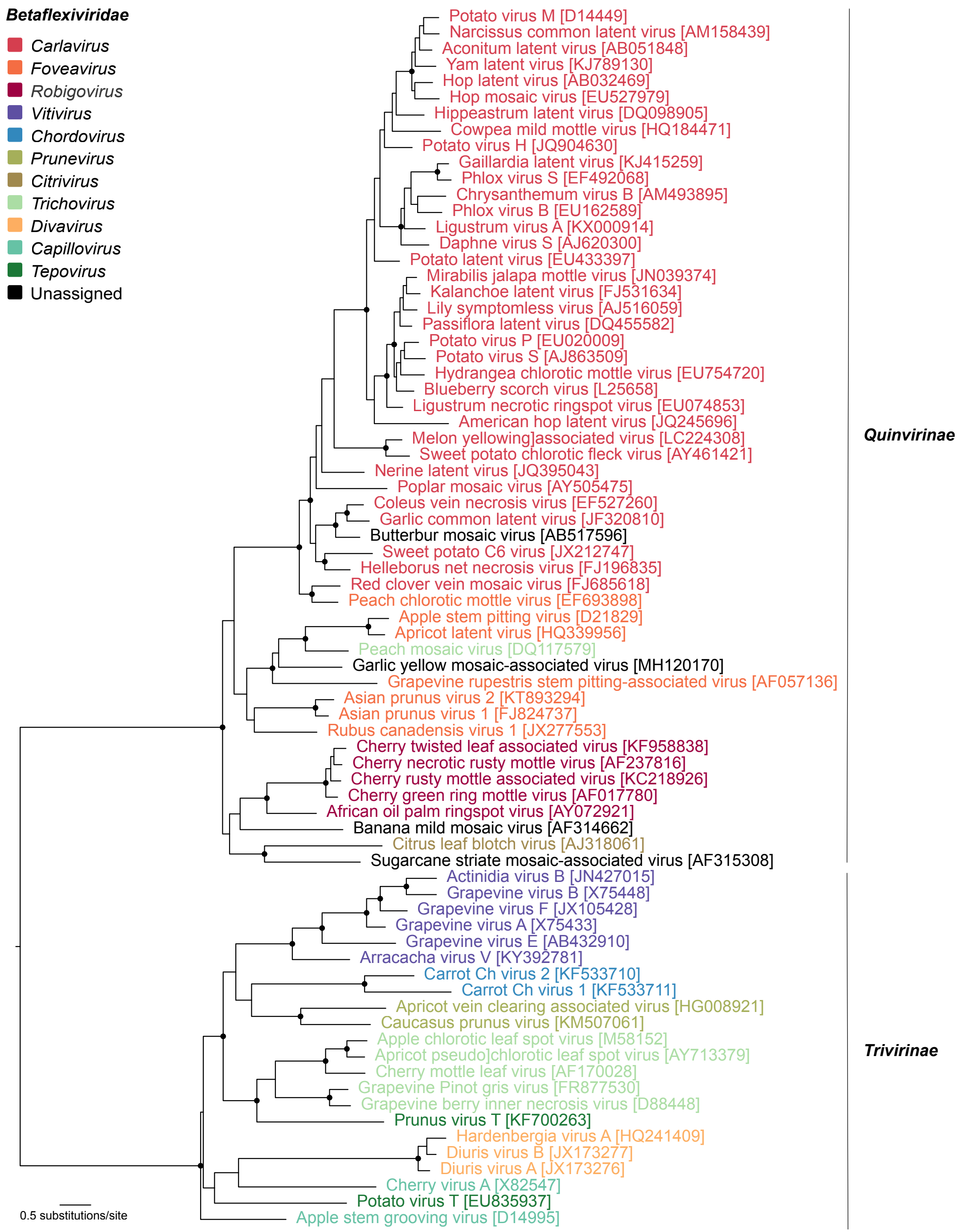
Betaflexiviridae
Carlavirus
Foveavirus
Robigovirus
Vitivirus
Chordovirus
Prunevirus
Citrivirus
Trichovirus
Divavirus
Capillovirus
Tepovirus
Unassigned
Potato virus M [D14449]
Narcissus common latent virus [AM158439]
Aconitum latent virus [AB051848]
Yam latent virus [KJ789130]
Hop latent virus [AB032469]
Hop mosaic virus [EU527979]
Hippeastrum latent virus [DQ098905]
Cowpea mild mottle virus [HQ184471]
Potato virus H [JQ904630]
Gaillardia latent virus [KJ415259]
Phlox virus S [EF492068]
Chrysanthemum virus B [AM493895]
Phlox virus B [EU162589]
Ligustrum virus A [KX000914]
Daphne virus S [AJ620300]
Potato latent virus [EU433397]
Mirabilis jalapa mottle virus [JN039374]
Kalanchoe latent virus [FJ531634]
Lily symptomless virus [AJ516059]
Passiflora latent virus [DQ455582]
Potato virus P [EU020009]
Potato virus S [AJ863509]
Hydrangea chlorotic mottle virus [EU754720]
Blueberry scorch virus [L25658]
Ligustrum necrotic ringspot virus [EU074853]
American hop latent virus [JQ245696]
Melon yellowing]associated virus [LC224308]
Sweet potato chlorotic fleck virus [AY461421]
Nerine latent virus [JQ395043]
Poplar mosaic virus [AY505475]
Coleus vein necrosis virus [EF527260]
Garlic common latent virus [JF320810]
Butterbur mosaic virus [AB517596]
Sweet potato C6 virus [JX212747]
Helleborus net necrosis virus [FJ196835]
Red clover vein mosaic virus [FJ685618]
Peach chlorotic mottle virus [EF693898]
Apple stem pitting virus [D21829]
Apricot latent virus [HQ339956]
Peach mosaic virus [DQ117579]
Garlic yellow mosaic-associated virus [MH120170]
Grapevine rupestris stem pitting-associated virus [AF057136]
Asian prunus virus 2 [KT893294]
Asian prunus virus 1 [FJ824737]
Rubus canadensis virus 1 [JX277553]
Cherry twisted leaf associated virus [KF958838]
Cherry necrotic rusty mottle virus [AF237816]
Cherry rusty mottle associated virus [KC218926]
Cherry green ring mottle virus [AF017780]
African oil palm ringspot virus [AY072921]
Banana mild mosaic virus [AF314662]
Citrus leaf blotch virus [AJ318061]
Sugarcane striate mosaic-associated virus [AF315308]
Quinvirinae
Actinidia virus B [JN427015]
Grapevine virus B [X75448]
Grapevine virus F [JX105428]
Grapevine virus A [X75433]
Grapevine virus E [AB432910]
Arracacha virus V [KY392781]
Carrot Ch virus 2 [KF533710]
Carrot Ch virus 1 [KF533711]
Apricot vein clearing associated virus [HG008921]
Caucasus prunus virus [KM507061]
Apple chlorotic leaf spot virus [M58152]
Apricot pseudo]chlorotic leaf spot virus [AY713379]
Cherry mottle leaf virus [AF170028]
Grapevine Pinot gris virus [FR877530]
Grapevine berry inner necrosis virus [D88448]
Prunus virus T [KF700263]
Hardenbergia virus A [HQ241409]
Diuris virus B [JX173277]
Diuris virus A [JX173276]
Cherry virus A [X82547]
Potato virus T [EU835937]
Apple stem grooving virus [D14995]
Trivirinae
0.5 substitutions/site
