# Supplementary material for: A new virus found in garlic virus complex is a member of possible novel genus of the family Betaflexiviridae (order Tymovirales)
Source: PeerJ. 2019 Jan 16;7:e6285. doi: 10.7717/peerj.6285 (PMC6339470; doi:10.7717/peerj.6285)
Supplement: Supplemental Information 1 — E-mail messages regarding submission of a taxoomic proposal of new plant virus genome sequence to ICTV. [file peerj-07-6285-s004.pdf]

Gmail

Bergmann Ribeiro <bergmann.ribeiro@gmail.com>

Taxonomic proposal

Murilo Zerbini <zerbini@ufv.br> 12 de novembro de 2018 08:58
Para: Bergmann Ribeiro <bergmann.ribeiro@gmail.com>

Dear Dr. Ribeiro,
Thank you very much for sending the Taxonomy Proposal proposing a new species in the family Betaflexiviridae. My initial assessment of the proposal is positive. I have forwarded it to the chair of the Beta-, Delta- and Gammaflexiviridae Study Group, Dr. Ioannis Tzanetakis, for further analysis. He will contact you in case there's anything to be revised. Otherwise, the TP will be considered during the upcoming 51th meeting of the ICTV Executive Committee.
Thank you again for your interest in viral taxonomy.
Best regards,
Murilo

On Thu, Nov 8, 2018 at 2:42 PM Bergmann Ribeiro <bergmann.ribeiro@gmail.com> wrote:

> **TP_Template_Word_module_2018_GYMaV.docx**
>
> Dear Professor Murilo Zerbini,
> I am sending you a taxonomic proposal for a new plant virus found in garlic plants. Attached to this message is an Excel file and a link for the taxonomic template. Could you please acknowledge receipt of this email, since I am submitting a manuscript to PeerJ and they require this information.
> Sincerely yours,
> Bergmann M Ribeiro
> --
> Bergmann Morais Ribeiro
> Prof. Titular,
> Pesquisador de Produtividade CNPq nível 1C
> Departamento de Biologia Celular
> Instituto de Ciências Biológicas
> Universidade de Brasília
> Brasília, DF, CEP 70910-900

--
Prof. F. Murilo Zerbini
Dep. de Fitopatologia
Universidade Federal de Viçosa

Viçosa, MG - Brasil
T: (+55-31) 3899-2935
F: (+55-31) 3899-2240
E: zerbini@ufv.br
